# Clinical translation of antibody drug conjugate dosing in solid tumors from preclinical mouse data

**DOI:** 10.1126/sciadv.adk1894

**Published:** 2024-05-31

**Authors:** Baron Rubahamya, Shujun Dong, Greg M. Thurber

**Affiliations:** ^1^ Department of Chemical Engineering, University of Michigan, Ann Arbor, MI 48109, USA.; ^2^ Department of Biomedical Engineering, University of Michigan, Ann Arbor, MI 48109, USA.; ^3^ Rogel Cancer Center, University of Michigan, Ann Arbor, MI 48109, USA.

## Abstract

Antibody drug conjugates (ADCs) have made impressive strides in the clinic in recent years with 11 Food and Drug Administration approvals, including 6 for the treatment of patients with solid tumors. Despite this success, the development of new agents remains challenging with a high failure rate in the clinic. Here, we show that current approved ADCs for the treatment of patients with solid tumors can all show substantial efficacy in some mouse models when administered at a similar weight-based [milligrams per kilogram (mg/kg)] dosing in mice that is tolerated in the clinic. Mechanistically, equivalent mg/kg dosing results in a similar drug concentration in the tumor and a similar tissue penetration into the tumor due to the unique delivery features of ADCs. Combined with computational approaches, which can account for the complex distribution within the tumor microenvironment, these scaling concepts may aid in the evaluation of new agents and help design therapeutics with maximum clinical efficacy.

## Teaser

Approved antibody drug conjugates drive tumor regression in mouse models at clinical doses due to similar uptake and distribution.

## INTRODUCTION

Antibody drug conjugates (ADCs) represent a rapidly growing class of therapeutics that includes six Food and Drug Administration (FDA)–approved agents for the treatment of patients with solid tumors. These drugs combine a potent small-molecule drug, most often a cytotoxic agent, connected to a tumor-targeted monoclonal antibody through a (typically cleavable) linker. Despite the recent success of ADCs, there is still significant attrition of these therapeutics during development (*1*). This is due to design complexities and multiple confounding factors that influence the effectiveness of these drugs. The potential for clinical success of an ADC is dependent on factors that may be antibody-specific, linker-specific, payload-specific, or target specific, but, most commonly, it is the unique combination of these factors for a particular target that determines the outcome (*2*). The complexity of design and lack of heuristics make it more challenging to predict whether an ADC under preclinical development will eventually be approved for use in the clinic.

One of the challenges in ADC development (or any drug) is scaling the results from preclinical studies for clinical development. In the predominant path, preclinical in vivo studies form an important bridge between in vitro assessment of candidates and first-in-human (FIH) trials, often using mouse models for efficacy. Despite their utility, xenograft mouse models, which are used for most preclinical in vivo studies, have known limitations going back many decades for small-molecule chemotherapeutics (*3*–*5*). Over the past 10 years, patient-derived xenograft (PDX) models are being more widely used to capture the variability found in clinical response (*6*). While these models help replicate differences in intrinsic payload sensitivity and target expression, they still suffer from the unique delivery challenges of ADCs. These limitations could result in screening out potentially successful drugs or the advancement of agents that eventually fail in the clinic. One important such limitation is that mouse models tolerate high (sometimes tumor-saturating) ADC doses that are often not translatable to humans. This is partly because of the discrepancy in normal tissue expression of ADC target antigens and typical lack of antibody cross-reactivity between humans and mice, thereby increasing the potential incidence of on-target off-tumor toxicities in clinical trials. More commonly, however, it is attributable to the fact that many ADC payloads are less potent in mouse versus human cells (e.g., Table 1), leading to greater normal tissue tolerability and relatedly, less tumor sensitivity in syngeneic models (*7*–*10*). For example, trastuzumab deruxtecan shows up to an order of magnitude less potency in human HER2-expressing mouse cells compared to a human breast cancer cell line [figure S4 of (*7*)], an EMT6–human HER2 (hHER2) mouse model is resistant to trastuzumab emtansine (*11*), and large doses of trastuzumab emtansine are needed in the HER2 Fo5 model (*12*). It is then possible (due to high tolerability) or necessary (due to syngeneic model low sensitivity) to dose at a much higher level than in the clinic. ADC doses that may be tumor-saturating and efficacious in mice often have to be administered at lower (sub-saturating) doses in the clinic that do not penetrate tumor tissue, do not reach all cancer cells, and may not show efficacy in humans.

**Table 1.** IC_50_ values of some ADCs/payloads in a human breast cancer cell line, HCC1954, a mouse breast cancer cell line, 4T1, and mouse E0771 cells stably expressing human HER2. Median inhibitory concentration (IC_50_) values in HCC1954 have previously been reported (*79*), while potency in 4T1 and E0771-hHER2 cells was measured according to the method in method S1. Apart from PBD, the ADC payloads show greater potency in the human cancer cell line than in the mouse cancer cell lines. CI, confidence interval; pM, picomolar; StDev, standard deviation.

	Human breast cancer	Mouse breast cancer	Ratio
	HCC1954 [IC_50_ in pM (± StDev)]	4T1 [IC_50_ in pM (95% CI)]	
**PBD**	49	43	0.9
	(±36)	(35 to 52)	
**Calicheamicin D**	3	79	26
	(±3)	(60 to 109)	
**MMAE**	37	1600	43
	(±29)	(1,400 to 1,880)	
**SN-38**	1,800	20,300	11
	(±820)	(12,000 to 179,000)	
**Exatecan**	750	2,874	4
	(±150)	(2,500 to 3,300)	
	**Human breast cancer**	**Mouse breast cancer**	
	**HCC1954**	**EO771-hHER2**	
**Trastuzumab emtansine**	19	37,000	1,900
	(±3)	(24,000 to 50,000)	
**Trastuzumab deruxtecan**	1,400*	>300,000	>200
	(±200)		
**Trastuzumab PBD**	44*	3.7	0.1
	(±3)	(3.3 to 4.3)	

*Measurements using trastuzumab with GlycoConnect conjugation to DXd (DAR 4) or PBD (DAR 2) in (*79*).

With several ADCs approved for the treatment of patients with solid tumors, this provides the opportunity to retrospectively examine the preclinical efficacy data in light of their clinical effectiveness. Notably, the FDA-approved compounds were able to show activity in some xenograft models when administered in a similar range of weight-based [milligrams per kilogram (mg/kg)] dose as the maximum tolerated dose (MTD) in humans. For a consistent definition, here “efficacy” is defined as having an equal or smaller tumor size than the starting size after 3 weeks (the most common dosing schedule in the clinic). Unlike small-molecule drugs, where a similar exposure, or area under the curve (AUC), is often desired to predict efficacy (*13*), these ADC studies suggest equal mg/kg dosing may better guide the evaluation of ADCs. Preclinical and clinical efficacy depend on a complex array of factors, so a single metric should not be used to replace dose-ranging studies, but this observation (efficacy after the same mg/kg dosing that is tolerated in the clinic) may help gauge the potential of ADCs during development. To support this guidance, we highlight data showing that a similar tumor concentration and tissue distribution of the ADC is achieved when given at the same mg/kg dose that is tolerated in humans, which provides a mechanistic explanation for this observation.

Given the proposed importance of matching the mg/kg dosing preclinically, this raises the question of how to best estimate this dose. We provide three possible strategies using current ADCs as a roadmap for clinical translation of dosing. More sophisticated computational models can help design efficacious ADCs and provide more accuracy by accounting for multiple nonlinear effects between animal models and humans, but the simplicity of this approach can be useful during early assessment. Therefore, this guidance may be an important reference point to aid the development of novel ADCs.

## PRECLINICAL EFFICACY OF APPROVED ADCs IN SOLID TUMORS

**Fig. 1.**
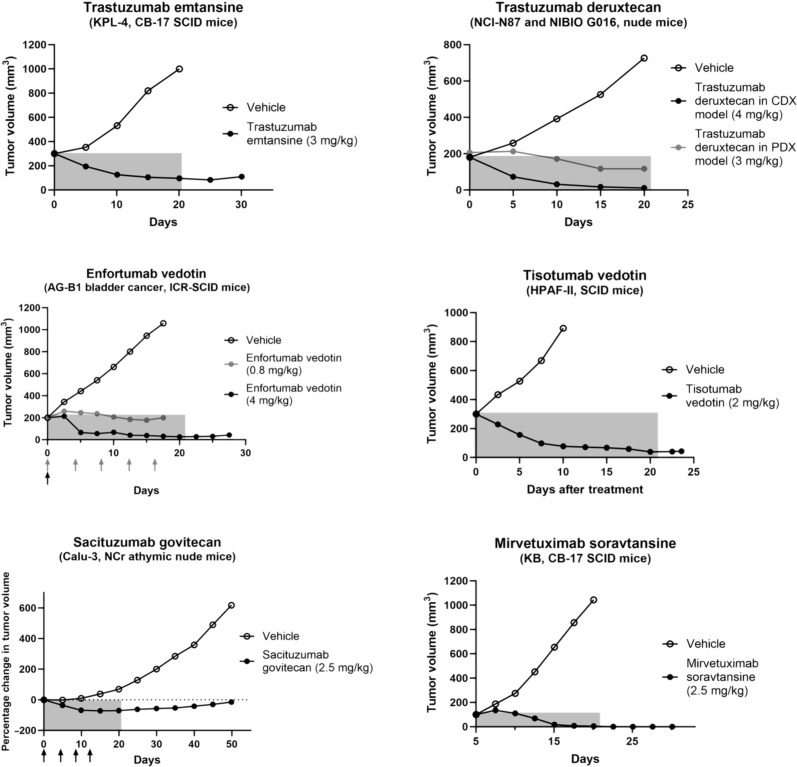
Efficacy of FDA-approved ADCs for solid tumors in preclinical mouse models. The efficacy of the six ADCs approved for treatment of solid tumor indications is shown from several published studies when the doses administered, in milligrams per kilogram (mg/kg), are close to the clinical mg/kg dose (*64*, *100*–*104*). Tumor growth curves were digitized from the indicated reference sources. The cell line and mouse strain used in each of the reported studies are shown below the ADC name. NIBIO G016 and AG-B1 PDX models were used to study the efficacy of trastuzumab deruxtecan and enfortumab vedotin, respectively. The clinical doses are 3.6 mg/kg Q3W for trastuzumab emtansine; 5.4 or 6.4 mg/kg Q3W for trastuzumab deruxtecan; 1.25 mg/kg on days 1, 8, and 15 of a 28-day cycle for enfortumab vedotin; 2 mg/kg Q3W for tisotumab vedotin; 10 mg/kg on days 1 and 8 of a 21-day cycle for sacituzumab govitecan; and 6 mg/kg Q3W for mirvetuximab soravtansine. SCID, severe combined immunodeficient.

Biologics are often administered using weight-based dosing (*14*), so we used published literature reports of the efficacy in mice when the mouse xenograft dose was matched to the clinical MTD (or was as close as possible to the clinical dose if the same dose was not available). We used the six ADCs approved by the FDA for the treatment of solid tumors as a benchmark, and Fig. 1 shows the efficacy following a single dose for these approved agents in select tumor models. (Exceptions to a single dose are indicated with arrows for the multiple doses with enfortumab vedotin and sacituzumab govitecan.) Notably, all six agents were able to decrease the tumor volume over the 3-week window (selected on the basis of the most common dosing interval used in the clinic; see figure caption for details, e.g., fractionated dosing for enfortumab vedotin). This indicates that the mg/kg dosing of an ADC for clinically approved agents is sufficient to reduce the tumor size at the end of a 3-week window in a mouse xenograft model before the next dose. Notably, the sensitivity of cancer cell lines can vary depending on the cellular ADC delivery properties, such as antibody target expression and internalization rate, along with the intrinsic potency of the payload in that cell line. These factors are discussed later, but, generally, it is assumed that xenograft models (cell line or PDX models) that mimic the expression and sensitivity of the target population are selected. Efficacy was seen with these models (but, notably, not all models) for each of the ADCs following a single-dose administration. In contrast, payloads like pyrrolobenzodiazepine (PBD) that have been successful in hematological cancers have not yet shown success in solid tumors. Xenograft models often require doses of PBD-based ADCs greater than the MTD for efficacy to be seen preclinically (*15*–*17*). Several additional examples of ADCs across different payloads and targets that have been discontinued require doses higher than clinically tolerable (*18*–*24*), contrary to the successful agents shown here.

To more thoroughly analyze discontinued ADCs, we examined the 92 discontinued compounds listed by Maecker *et al.* (*1*) for preclinical and clinical results. Of these, 54 ADCs tested in solid tumors had data suitable for analysis, with 37 having reported both preclinical data and clinical MTD values. Because dosing regimens vary widely in this retrospective analysis (typically using more frequent dosing in mice than humans), we aggregated the doses in mice over the 3-week window (typical dosing cycle in humans) if single-dose studies were not available to best leverage the data. For example, a 2.5 mg/kg dose every 4 days for four doses (2.5 mg/kg Q4Dx4) in mice was considered a 10 mg/kg dose compared to the 1.88 mg/kg every 3 weeks (1.88 mg/kg Q3W) in humans. Of the 37 discontinued ADCs examined, only 7 had data showing efficacy as defined above (tumor shrinkage over 3 weeks in at least one mouse model) at or below the clinical MTD (table S1). Of the 17 agents with incomplete data, only 2 of these showed efficacy at or below the highest dose reported in the clinic. A notable limitation of this analysis is that many ADCs only evaluated efficacy at much higher doses and/or multiple doses in mice. However, some of these agents required substantially higher doses in mice than the clinical MTD to see sufficient efficacy [e.g., 36 mg/kg of total ADC versus a 3.6 mg/kg MTD; (*25*, *26*)].

For the nine discontinued agents that showed efficacy in mice at or below either the clinical MTD or highest tested clinical dose, these all had measurable clinical activity. Two of them targeted HER2 including SYD985 (*27*, *28*) with a 33% partial response (PR) rate. XMT-1522 was discontinued for strategic reasons related to competitiveness in the HER2 space before reaching the MTD but showed stable disease (SD) or better in 85% of patients with a PR at the higher doses. The other agents include DMUC4064A (with a 39% PR/CR rate and 35% SD) (*29*, *30*), DLYE5953A (12% PR and 54% SD) (*31*, *32*), DEDN6526A (11% PR and 32% SD) (*33*, *34*), SAR566658 (13% PR and 39% SD), ADCT-401 (12% composite response rate for PSA with 3% PR and 36.4% SD), and RN927C (37.9% SD although not fully explored due to toxicity) (*35*, *36*). Other factors, such as trafficking and linker release, may play a role in efficacy of these ADCs (*37*, *38*). The final agent, enapotamab vedotin, showed PRs in three patients, but it is unclear how many were treated at these higher dose levels. Notably for this last agent, the only model that showed efficacy at or below the clinical MTD was the sole CDX model, while the nine PDX models were all dosed at four to eight times higher in mice (and above the clinical MTD).

There are many factors involved in drug approval beyond just dosing and response rates, including patient, medical, and industry considerations such as toxicity/side effects/quality of life, comparisons with the current standard of care, and drug pipeline prioritization. Given the complexity of ADC design, there are both discontinued agents that have demonstrated efficacy at or below the clinical MTD and ADCs that have not published data showing this level of efficacy in mice but have shown clinical responses [e.g., BAT8001 with a 41% PR and 83% disease control rate; (*39*)]. Because efficacy is a major driver in these decisions, these results highlight how a larger proportion of approved drugs have demonstrated preclinical efficacy at or below the clinical MTD versus those that are discontinued. In summary, a higher percentage of approved ADCs have demonstrated efficacy in at least one mouse model at or below the clinical mg/kg MTD than discontinued agents, with 6 of the 6 approved agents (100%) and 7 of the 37 discontinued agents (19%) having published ADC activity. Of these 19% of discontinued agents, all had measurable clinical activity (CR, PR, and SD responses).

This observation raises the question as to why, from a mechanistic standpoint, matching the mg/kg dose in mice might resemble the needed mouse efficacy for translation to clinical efficacy. Clearly, the distribution and responses are more complicated than what is captured in these animal models, and many groups, including our lab, use more sophisticated simulations to scale from preclinical to clinical results (*40*–*42*). However, simple heuristics can be useful guides to evaluate the utility of different compounds and check the reliability of alternative scaling approaches. For this approach, there are two main reasons why the mg/kg dosing correlates with response: tissue penetration and payload concentration.

### Tissue penetration

Recent evidence suggests that the penetration of ADCs into solid tumors is a critical parameter in determining clinical efficacy (*41*, *43*). Tissue penetration refers to the distance the ADC, and the payload, in particular, is able to reach beyond the blood vessel from which it extravasates within the tumor. Because of the rapid binding of antibodies relative to their transport through tissue, often mediated by diffusion due to elevated interstitial pressure, antibodies (and ADCs) must saturate the perivascular cells before penetrating deeper into the tissue. As this occurs, the cancer cells internalize and degrade the ADC, not only releasing the payload but also consuming the ADC before it can reach deeper in the tissue. Therefore, the concentration of ADC needed to saturate these perivascular cells, rather than the total exposure (AUC), is the major factor in determining the maximum tissue penetration distance. The perivascular distribution of the biologic, often referred to as the “binding site barrier” is well-established in both animal models and the clinic due to the rapid binding rates relative to diffusion (*44*–*53*). These factors have been shown to be important for multiple modalities by controlling access of the therapeutic to its site of action (*54*–*56*).

**Fig. 2.**
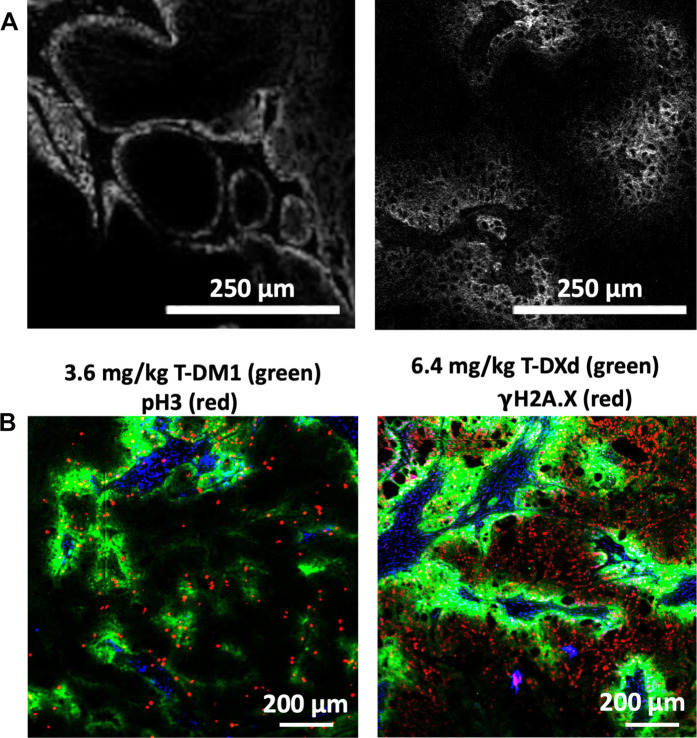
Tissue penetration of antibodies and ADCs in mice and humans. The high affinity anti–epidermal growth factor receptor (EGFR) antibody, panitumumab, penetrates only a few cell diameters in human patients with head and neck cancer when administered at a 1 mg/kg dose used for intraoperative imaging (**A**, left) (*53*). Likewise, the anti-EGFR antibody, cetuximab, shows a similar tissue penetration following a 1.2 mg/kg dose in a mouse xenograft model (A, right) (*60*). Tissue penetration can be increased by multiple mechanisms including higher antibody doses from the coadministration of unconjugated antibody, lowering the drug-to-antibody ratio (DAR) so a higher ADC dose is tolerated, or the use of lower potency payloads. In the case of trastuzumab emtansine versus trastuzumab deruxtecan, the lower potency topoisomerase inhibitor allows trastuzumab deruxtecan to be administered at 6.4 mg/kg, increasing the tissue penetration of the ADC labeled in green (**B**). In addition, the payload on trastuzumab emtansine does not exhibit bystander effects, meaning it cannot exit ADC targeted cells to reach untargeted cells. A pH3 marker of cells trapped in mitosis (red) only overlays with the trastuzumab emtansine labeled cells (B, left). In contrast, the DXd payload can diffuse out of targeted cells. While this does not completely overcome incomplete tissue penetration, the DXd payload released from trastuzumab deruxtecan (B, right, green) is able to cause some DNA damage in cells untargeted by the ADC as seen by the γH2AX stain (red).

Administering similar mg/kg dosing scales the dose per body weight, and because many species have a similar blood volume per body weight (*57*), this matches the (initial) plasma concentration between species after an intravenous infusion. Because tissue penetration is proportional to plasma concentration [which can be represented by a Thiele modulus; (*58*, *59*)], matching the mg/kg dosing in mice matches the maximum tissue penetration in the clinic (*53*, *60*). By administering the same mg/kg dosing in preclinical models, the tissue penetration and, therefore, the relative number of cells per tumor volume reached by the therapeutic are similar to the clinic (Fig. 2A). While ethical factors and business considerations often preclude head-to-head comparisons of different formats in the clinic, an interesting example can be seen with two agents targeting MUC16. With the same antibody and same payload (albeit different conjugation sites), the lower drug-to-antibody ratio (DAR) thiomab format enabled over twice the clinical dose of antibody (with a similar total payload dose), which resulted in over twice the clinical response rate (fig. S1) (*30*, *61*). Unfortunately, the thiomab agent also showed an increase in ocular toxicity precluding further development. However, this improved efficacy is consistent with preclinical and clinical data showing heterogeneous distribution (*44*–*53*) and improvements in efficacy with higher antibody dosing/tissue penetration (*54*, *62*–*65*).

The factors influencing growth rate (implantation site, cell doubling time, etc.) generate variability in efficacy studies, so a single value should not be used for evaluation. Hence dose-ranging studies and tests in multiple models are necessary. However, these results suggest that efficacy studies near the clinical MTD can be used to gauge potential. For example, an agent demonstrating preclinical efficacy at a dose that is 2-fold higher than the clinical MTD might be judged to have more potential, all other things equal, than an agent requiring a 10-fold higher dose than the clinical MTD for preclinical efficacy, where the latter result could be used to motivate alternative design parameters to improve efficacy (modifying DAR, linker, payload, etc.).

Aside from the plasma concentration, the exposure (or AUC) is another pharmacokinetic factor used in evaluation. The immunoglobulin G (IgG) backbones of ADCs typically result in long half-lives, enabling a pseudo-steady state to develop between the blood and tumor tissue, albeit with a much lower tumor concentration than the plasma concentration due to transport limitations (*58*). (Exceptions include ADCs where the AUC may be affected by a fast clearance rate, for example, due to an unstable linker, which could reduce tumor penetration.) If an ADC is well tolerated and dosed high enough to achieve efficient tissue penetration on account of its high plasma concentration, then the impact of AUC is reduced. In contrast, if a larger preclinical ADC dose is given to match the exposure (AUC) by compensating for faster clearance, then the tissue penetration will be significantly higher than what can be achieved in the clinic and could overestimate efficacy.

In addition to the ADC tumor distribution, bystander cell killing can also affect tissue penetration of the payload and must be considered. Bystander effects account for payload that either diffuses out of targeted cells or is released from the ADC extracellularly, and this payload may also contribute to payload tissue penetration and efficacy. However, previous work has shown that intracellular ADC payload delivery is more efficient at killing cells compared to bystander killing (*42*). While it has been hypothesized that free payload released from ADCs in circulation is important for ADC efficacy (*66*), the magnitude of this effect relative to antibody-mediated delivery is unclear. Many DAR 4 Monomethyl auristatin E (MMAE) ADCs with the same linker chemistry but targeting different tumor antigens have been developed, but only a limited number have shown utility in the clinic. In addition, although a substantial amount of payload is released from sacituzumab govitecan during circulation (*67*), resistance of patients to the ADC has been reported because of loss of tumor expression of the TROP-2 target (*68*). These data suggest that free payload likely provides some uptake but not enough to drive significant efficacy without additional more efficient release from targeted ADCs.

Improved tissue penetration can also be seen when comparing trastuzumab deruxtecan versus trastuzumab emtansine in mouse tumor models. The greater tolerability of the topoisomerase inhibitor allows higher dosing at 5.4 or 6.4 mg/kg versus 3.6 mg/kg with the microtubule inhibitor. While higher ADC penetration is more effective than bystander killing, the bystander payload can still diffuse to deeper cells, further improving the tissue penetration of the trastuzumab deruxtecan payload (Fig. 2B). The methods used to study intratumoral ADC distribution and pharmacodynamic immunofluorescence staining are detailed in method S2. The higher tissue penetration of the ADC and payload may contribute to the higher efficacy seen with trastuzumab deruxtecan versus trastuzumab emtansine.

### Payload concentration

**Fig. 3.**
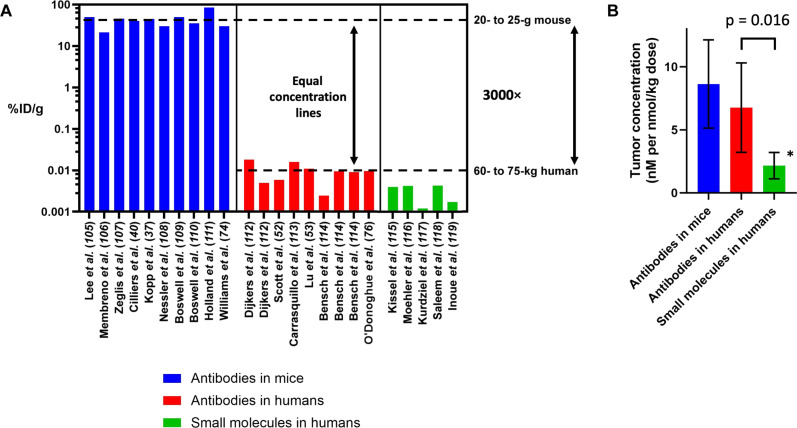
Antibody and payload uptake in mouse and human tumors. Literature reports of antibody uptake in mouse tumors are often around 30%ID/g depending on factors such as vascular density/necrosis and blood vessel permeability, with several typical values shown here (*37*, *40*, *74*, *105*–*111*). Because of the larger body weight of humans, the clinical uptake of antibodies, estimated from molecular imaging and biopsy samples, is ~0.01%ID/g, as seen with several typical values calculated from contemporary studies (*52*, *53*, *76*, *112*–*114*) (**A**). However, this results in a similar concentration of antibody in the tumor when administered at the same mg/kg dose (**B**). Clinical estimates of small-molecule drugs are also available from molecular imaging experiments, and the concentrations are typically threefold lower than the concentration of payload delivered by an ADC (*115*–*119*). In addition, the concentrations drop much faster for small-molecule chemotherapeutics, providing a therapeutic advantage for ADCs (*maximum concentration shown before rapid drop). Methods used to calculate concentrations are shown in methods S3 and S4.

Besides tissue penetration, the second reason for using the same mg/kg dosing in mice is to achieve a similar ADC concentration within the tumor. Assuming efficient linker cleavage and payload release for these agents [where linker and payload dose are important design factors; (*69*, *70*)], the ADC concentration drives the intratumoral payload concentration. This is the ultimate mediator of drug effect, where the payload concentration drives mass-action kinetics of binding to the payload target and disruption of a biochemical process (*71*). Often, a minimum payload concentration is needed to drive sufficient response (*71*–*73*). From radiological precedent, the units used to measure drug uptake are often percent of injected dose per gram of tissue (%ID/g), which are convenient for quantifying radiolabeled compounds by dividing by a reference dose to account for radioactive decay. However, it is important to note that the %ID/g units are extrinsic and, therefore, scale with body weight (*74*), while concentration is intrinsic (and independent of body weight). Put another way, the same concentration of drug in a tumor will result in ~3000-fold lower %ID/g in humans than mice (assuming a 60- to 75-kg human versus 20- to 25-g mouse). Figure 3A shows several representative values of %ID/g of antibodies in clinical tumors versus mice. When accounting for the differences in body weight, a similar intrinsic amount of antibody or antibody conjugate (i.e., concentration) reaches mouse versus human tumors. Therefore, from a drug efficacy standpoint, it is true that a small fraction of the ADC ends up in the tumor. However, the concentration of drug achievable in a human tumor is actually similar to tumors in mice when dosed at a similar mg/kg level (Fig. 3B). Mechanistically, this observation is predicted because antibody/ADC uptake into tumors is driven by the vascular permeability and surface area, resulting in similar total uptake for antibody-based therapeutics when given at sub-saturating doses (*58*). Therefore, the theoretical, preclinical, and clinical data are all in agreement.

This analysis also provides the opportunity to compare ADCs with small-molecule chemotherapeutics. Colombo and Rich (*75*) recently demonstrated that current ADCs do not significantly increase the MTD of the payloads. In other words, once the payload mass of an ADC is taken into account, a similar dose of the small-molecule (payload) is tolerated whether it is conjugated to an antibody or not. This is consistent with the observation that most of the ADC/payload is metabolized elsewhere in the body rather than the tumor, supporting the idea that a similar amount of payload would be delivered to healthy tissue in both cases.

Colombo and Rich (*75*) also noted that, although the tolerability of a small-molecule was similar whether administered as a free drug or attached to an antibody, the efficacy of the ADCs was much higher. This could be due to more efficient delivery at the macroscopic (organ/tumor) or microscopic (cellular) level. From an organ/tumor-level biodistribution analysis, Fig. 3A shows the %ID/g of small molecules in humans at peak uptake. On average, these small molecules achieve ~4%ID/kg in the tumor, which is threefold lower than antibodies/ADCs. In addition, the peak values for small molecules occur shortly after administration (~10 min) followed by a rapid decrease, so this transient concentration may not be able to fully engage the target. In contrast, data from theoretical calculations, mouse, and clinical results show the highest antibody uptake occurs over the first 1 to 3 days followed by a slow decrease (*73*, *76*, *77*). Assuming linker stability over this time [which is true for most ADCs with the exception of sacituzumab govitecan; (*37*, *70*)], ADC uptake would be similar to these antibodies. Accounting for the molecular weight differences, Fig. 3B shows that a higher tumor concentration is achievable with antibodies when administering the same number of payload/small molecules. Therefore, the tumor has a higher level and greater exposure to the payload from an ADC than a small-molecule chemotherapeutic (*78*). Furthermore, at the cellular level, ADCs release the payload inside targeted cells, which is more efficient than bystander killing, providing an additional advantage from the subcellular proximity to the payload target (*42*, *79*). At the tissue level, however, there is a significant caveat. Small-molecule drug gradients dissipate quickly relative to ADCs (*80*), ensuring efficient tissue penetration for most small molecules. This is a major potential disadvantage for ADCs if the tissue penetration is not taken into account. Therefore, ADCs have a delivery advantage over small molecules if they are able to efficiently penetrate the tumor and deliver sufficient payload per cell.

Despite the well-known differences in pharmacokinetics between mice and humans, this analysis indicates that ADCs may scale better to the clinic by using equivalent mg/kg dosing (*81*) rather than directly normalizing for plasma clearance. Other methods can provide additional input, such as more sophisticated computational modeling that can provide more accurate estimates by accounting for the multiple factors involved, the use of multiple animal models to capture variability in expression/trafficking/response, and dose escalation studies to provide a more comprehensive view on the necessary dose for efficacy. However, this simple criterion can provide a straightforward and early gauge of potential from animal efficacy data.

## TOLERABILITY OF CLINICAL ADCs

The analysis above indicates that it is important to evaluate the efficacy of an ADC in mouse models when administered at the clinical MTD because of similar tissue penetration and tumor concentration at these doses. However, this raises the conundrum of how to determine the clinical MTD before FIH studies. There are three current approaches that provide promise depending on what, if any, clinical data are available: (i) when other clinical ADCs with the same payload have been reported, (ii) when other clinical small molecules similar to the payload have been reported, or (iii) if neither case exists due to the novelty of the payload. These approaches are meant to complement the dose estimation in mice for early evaluation rather than the full suite of data needed to proceed with FIH clinical trials.

The toxicity of ADCs is generally associated with their cytotoxic payloads, limiting their MTD, whereas most monoclonal antibodies are well tolerated and can be dosed in relatively large amounts. Although exceptions exist for any simple rule [e.g., Ephrin type A2 receptor (EphA2) antibodies inducing clotting effects (*82*), strong Fc-effector function (*83*), and/or signaling in healthy tissue (*84*)], a majority of ADC toxicity stems from the cytotoxic payload. MTDs determined in mouse models during preclinical studies are often not an accurate depiction of human ADC tolerability; mouse MTDs are typically much higher than tolerability in humans, even when scaling based on body surface area. This stems from two major factors: differences in payload potency between species and differences in antibody target expression and cross-reactivity of the antibody. Therefore, previous clinical data and/or nonhuman primate (NHP) data are most useful for determining tolerability when available.

The discrepancy in tolerability due to differences in potency between species has been reported for important ADC payloads including auristatins, PBDs, and camptothecins. For example, studies with dolastatin-10, the parent molecule of MMAE, showed that mice tolerated much higher doses of this cytotoxin compared to the human MTD (*85*), and mice have shown lower sensitivity to topoisomerase inhibitors compared to humans (*9*). In such cases, mouse models would not be an accurate method for assessment of drug tolerability. Whereas rats and NHPs are the species most commonly used to study the toxicity of ADCs, rats are used less frequently than mice to study ADC efficacy. The current analysis indicates that doses in mice need to match tolerability in humans, which can, in turn, be estimated by a variety of species/techniques as highlighted here. Without accounting for these differences, ADCs that show promise during preclinical development at high doses tolerated in mice may fail in human trials because of unacceptable toxicity. The first ADC to obtain FDA approval, Mylotarg, is a notable example of a toxicity-related market withdrawal (*86*). The ADC subsequently gained reapproval at a lower, more tolerable dose. Critical evaluation of dosing strategies is therefore required to be able to determine preclinical MTDs that are representative of ADC tolerability in the clinic. Because a majority of the ADC is metabolized outside the tumor due to nonspecific processes such as macropinocytosis (*87*) or linker cleavage in circulation, the sensitivity of human tissues to the payloads and the amount taken up by certain cells are important for assessing tolerability. Therefore, the best data on ADC tolerability are from previous clinical studies.

### Approach 1: Clinical ADC data

Previous reports (*88*, *89*) have shown that the toxicities associated with ADCs bearing the same payloads are often similar, irrespective of the antigen bound by the antibody backbone. For example, ADCs with MMAE payloads generally induce periphery neuropathy, while Monomethyl auristatin F (MMAF) and DM4 (a maytansine derivative) ADCs cause ocular toxicity. The MTDs of ADCs under preclinical development can therefore be expected to be similar to those of previously clinically tested ADCs with the same payload, DAR, and linker chemistry. The MTDs of DAR 4 MMAE ADCs are typically about 1.8 mg/kg, irrespective of the ADC’s target antigen because the total payload dose delivered is the same, therefore mediating similar toxicity. In addition, the MTDs of DAR 2 MMAE ADCs have been reported at about double the dose (3.6 mg/kg) (*90*), consistent with the total payload dose, not the antibody dose, driving toxicity. This is also the case with DAR 4 deruxtecan (DXd) ADCs whose MTDs are approximately twice those of their DAR 8 counterparts (*75*, *91*).

The ability to administer higher antibody doses with a lower average DAR, either directly or by coadministration of unconjugated (“DAR 0”) antibody, has been shown to improve efficacy by increasing tissue penetration (*40*, *64*). Therefore, to achieve tumor saturation and direct cellular drug delivery, which are associated with improved efficacy, it is often better to lower the DAR of an ADC with a sub-saturating dose to increase the total antibody administered to a dose that penetrates farther into the tumor and directly targets more cells (*40*). Because it is easier to saturate healthy tissue, a higher dose of a lower DAR ADC (or the coadministration of a DAR 0 antibody dose) will often supersaturate the healthy tissue, resulting in ADC washout and delivery of less cytotoxic payload to healthy tissue expressing the target, providing another reason for lower DAR. In contrast, if the clinically tolerated ADC dose saturates the tumor and all the tumor cells are targeted, then a higher DAR will increase the amount of payload delivered per tumor cell and would increase efficacy (*92*). Therefore, the optimal DAR is dependent on the clinical MTD and the target expression and internalization rate (that determine the tumor saturating dose).

Whereas ideal ADC target antigens would be completely tumor specific, many ADC targets, like TROP2 (*93*), are expressed by normal tissue at levels that may drive significant target-mediated toxicity, resulting in on-target off-tumor toxicity. In these cases, the MTD may be dependent on the antibody target, unlike the scenario discussed above. Consideration therefore must be made to account for the possibility of target-mediated toxicity, depending on the target chosen. For example, trastuzumab deruxtecan, a DAR 8 ADC that targets HER2, is tolerated at a clinical dose of 5.4 or 6.4 mg/kg, so a DAR 4 DXd ADC could in theory be dosed around 10.8 to 12.8 mg/kg. However, the clinical MTD of the DAR 4 DXd ADC datopotamab deruxtecan was determined to be 8 mg/kg (*94*), partly because of toxicity mediated by TROP2 expression by normal tissue. On the other hand, DS-7300, a DAR 4 ADC that targets B7H3 (*95*), has a recommended phase expansion dose of 12 mg/kg (*91*) as its target antigen has limited expression in normal tissue (*96*). Antibodies that are cross-reactive to NHP antigens can be used in these circumstances to help assess antibody target-mediated toxicity as described under Approach 3 below.

### Approach 2: Clinical small-molecule data

In the absence of a precedent ADC bearing the same payload, ADC MTDs can be estimated on the basis of the clinical tolerability of the payloads or related cytotoxins when used as standalone chemotherapy. When normalized for conjugated payload, Colombo and Rich (*75*) demonstrated a similar MTD between small-molecule drugs administered alone or as an antibody conjugate. This approach can leverage previous clinical data before conducting the extensive toxicity studies needed for FIH dosing.

Similar to the use of MTDs from ADCs with the same payload, this approach will not capture target-mediated uptake of the ADC. While previous reports (*88*, *89*) have highlighted how many ADCs have dose-limiting toxicities unrelated to the antigen, this assumption will always need to be tested at a later stage. To guide the dosing in mouse models for measuring efficacy, however, this approach provides another avenue to use clinical data.

### Approach 3: NHP data

Estimations of FIH doses and human MTDs can also be made on the basis of the highest non-severely toxic dose (HNSTD) in NHPs (*89*). More extensive analysis is needed to scale animal results to the clinic for FIH dosing, but the intent here is to estimate the clinical tolerability to help scale the preclinical efficacy studies earlier in the drug development pipeline. Using the human MTDs of FDA-approved ADCs and their reported HNSTDs in cynomolgus monkeys, clinical MTDs are found to be two- to sixfold lower than the cynomolgus monkey HNSTD on a body weight basis (Table 2). The NHP data have the advantage of being able to examine target versus non–target-mediated toxicity given the much high prevalence of antibody cross-reactivity between human and NHP proteins on account of the similarity of their antigen sequences. These data can also be used to examine the potential for target-mediated drug disposition in a cross-reactive species (*97*). In contrast, many antibodies against human targets are generated by immunizing mice, so these antibodies are unlikely to cross-react in the preclinical mouse models used for efficacy studies since tolerance induced during B cell development regulates the incidence of antibodies that are autoreactive to mouse epitopes.

**Table 2.** Highest non-severely toxic dose in cynomolgus monkeys versus the clinical dose of approved ADCs in solid tumors. Values in parentheses indicate the cumulative doses of enfortumab vedotin and sacituzumab govitecan over a 21-day cycle as well as the corresponding ratios of HNSTD to clinical dose.

ADC	HNSTD in cynomolgus monkeys (mg/kg)	Clinical dose (mg/kg)	Ratio of HNSTD to clinical dose
Trastuzumab emtansine	10	3.6	2.8
Trastuzumab deruxtecan	30	6.4	4.7
Enfortumab vedotin	3	1.25* (3.75)	2.4 (0.8)
Sacituzumab govitecan	60	10† (20)	6 (3)
Tisotumab vedotin	3	2	1.5
Mirvetuximab soravtansine	10	6	1.7

*Day 1 (D1), D8, and D15 of a 28-day cycle.

†D1 and D8 of 21-day cycle.

### Perspectives and limitations

The analysis presented here has several limitations and considerations. Similar to Lipinski’s rule of five for small molecules (where violations are used for further scrutiny rather than elimination), a lack of efficacy alone in mouse models at the clinical MTD should not be used to abandon an ADC. There are many animal models that have intrinsic resistance to a payload, for example, and target expression levels can vary between models and the clinic. In addition, some mouse tumors grow extremely rapidly, which can affect the potency and efficacy relative to clinical tumors, particularly when efficacy is measured 3 weeks after treatment. Notably, several discontinued ADCs that did not publish efficacy in mouse models at or below the clinical MTD showed clinical responses. However, a lack of efficacy at these doses raises a “flag” that should be understood in more detail before proceeding. Likewise, success in an animal model at the clinical MTD is no guarantee of overall clinical approval as exemplified by the seven discontinued ADCs that had efficacy at the clinical MTD. Many factors, such as tolerability/patient quality of life, efficacy versus the current standard of care, and business strategies play a role. However, efficacy is often a central feature during evaluation. Other toxicities [such as bleeding observed for MEDI-547; (*82*)] not seen in mice, highly sensitive cell lines, or expression from transfected cell lines above clinical levels can skew the results in a positive direction. Multiple PDX models provide a broader overview of clinical sensitivity and expression differences, and these are often used later in the preclinical pipeline. A larger dataset testing the clinical MTD in PDX models for the approved agents would help refine the estimates based on preclinical models. However, given the tendency of other estimates to yield excessively optimistic projections, such as therapeutic windows in the 10s to 100s that ultimately result in very narrow (if any) clinical window, this simple measure shown for approved agents can provide a path forward to gauge the therapeutic potential based on animal studies designed to mimic targeting in the clinic.

Another limitation from animal models is the lack of immune effects, which can affect both toxicity and efficacy. Trastuzumab deruxtecan showed negligible activity in an immunodeficient mouse bearing a syngeneic tumor (likely due to lower payload sensitivity of the mouse tumor cells), but the same tumors showed significant response in an immunocompetent mouse (*7*). It is therefore expected, consistent with the literature (*98*), that immune effects may contribute to the efficacy of ADCs in immunocompetent mouse models, which is not captured in this measure of efficacy based on immunodeficient models. Likewise, these effects can cause unexpected toxicity even beyond what NHP data can show. This can be particularly acute for immune-stimulating payloads such as Toll-like receptor or STING agonists. Therefore, this approach is most appropriate for monoclonal antibodies with well-studied cytotoxic payloads. It is also important to note that the examples presented here are all IgG-based formats. Because of their size and Fc domains, the terminal half-lives in mice are still several days, providing sufficient exposure for tumor uptake. The scaling rules are unlikely to apply to smaller molecule formats due to their faster plasma clearance (in both mice and humans) and the often-better tissue penetration achieved.

Despite the emphasis on tissue penetration and payload concentration (versus exposure or AUC), the full drug disposition of ADCs is complex, and continuous delivery of payload over an extended period of time can play a role in certain conditions. This again highlights the challenges in ADC design and the continued need for more sophisticated computational approaches. Previous analysis has demonstrated how a similar total antibody dose (that determines tissue penetration) but different DARs can yield similar efficacy (*42*). This is because the same fraction of cells is “reached” by the ADC in both cases, and the different concentrations of payload are both still sufficient for cell death, resulting in similar efficacy. In other words, the tissue penetration of the ADC can outweigh the impact of total payload delivered. However, if the amount of payload being delivered is close to the minimum needed to kill the cells, then a more sustained exposure can be beneficial in those cases. For example, in (*29*) (see Fig. 3B), a fairly resistant xenograft (with minimal growth reduction at 6 mg/kg) had a better response to a thiomab with a more stable linker, thereby increasing the exposure. This is in contrast to the more sensitive cell lines, where the total ADC dose (independent of the payload dose) drove efficacy. Therefore, neither the maximum concentration (*C*_max_) nor the AUC is by itself sufficient for scaling ADCs from mice to humans. In practice, *C*_max_ may dominant under many experimental conditions. One reason for this is that, even in mice, ADC concentration is maintained over a few days, providing sufficient time/exposure for cell killing; *C*_max_ helps determine the number of cells that are exposed/killed. A long exposure at low concentrations cannot kill many cells because the ADC cannot reach cells deeper in the tumor. This is why fractionated dosing (when administered at sub-saturating doses) is generally less effective than a large bolus dose that can reach more cells (*99*) (e.g., a single 4 mg/kg dose of enfortumab vedotin is more effective than 5 doses at 0.8 mg/kg in Fig. 1). Measurements of *C*_max_ can vary depending on experimental conditions such as how soon the first time point measurement is taken. Because antibodies typically distribute in the plasma volume quickly and plasma volumes per body weight are similar, mg/kg dosing was used as a surrogate for *C*_max_ in this analysis. While the emphasis here was on evaluating efficacy at clinically relevant doses, the complexity of ADC delivery requires more detailed simulations and analysis for more accurate predictions on ADC modifications during development. This highlights the strength of joint experimental and computational approaches to design more effective agents.

## CONCLUSIONS

In this work, we highlight how ADC efficacy in mouse models, when the drug is administered at a similar mg/kg dose as tolerated in the clinic, correlates with clinical efficacy and FDA approval status. Insight into clinical potential can be gained by determining the ADC efficacy at or near the clinical MTD in mouse models. By using weight-based dosing in mice, the tissue penetration and payload concentration are similar to clinical reports, providing a mechanistic justification for the approach. The complexity of ADC design necessitates the use of multiple assessments of efficacy and toxicity, but these come at the cost of significant time and resources. Therefore, early evaluations of clinical potential by using clinically relevant dosing, such as the one described here, can be used to help streamline the ADC design and identify the most promising candidates.
