# Supplementary material for: Clinical translation of antibody drug conjugate dosing in solid tumors from preclinical mouse data
Source: Sci Adv. 2024 May 31;10(22):eadk1894. doi: 10.1126/sciadv.adk1894 (PMC11141632; doi:10.1126/sciadv.adk1894)

Science Advances
AAAS

Supplementary Materials for

Clinical translation of antibody drug conjugate dosing in solid tumors from preclinical mouse data

Baron Rubahamya *et al.*

Corresponding author: Greg M. Thurber, gthurber@umich.edu

Sci. Adv. **10**, eadk1894 (2024)
DOI: 10.1126/sciadv.adk1894

This PDF file includes:

Table S1
Methods S1 to S4
Fig. S1

Supplemental Table S1:

54 discontinued ADCs tested in solid tumors that were analyzed for preclinical and clinical results.

Of the 54 discontinued ADCs in solid tumor indications with data available including 37 for which preclinical and clinical data was published, 7 showed efficacy (tumor regression over a 3-week clinical dosing interval) in mice when administered at or below the clinical MTD (in mg/kg dosing). There were 4 other ADCs that we did not include in the count of 7 that warrant further discussion. Sofituzumab vedotin and PCA062 both showed tumor stasis over the dosing window (e.g., regression but regrowth reaching the initial tumor size). MDX-1203 did not show tumor growth curves, but the publication indicated efficacy starting at 4 mg/kg (versus 8 mg/kg clinical MTD). However, complete responses in mice required 2 doses of 38 mg/kg, so it is assumed the 4 mg/kg activity was able to slow growth but not achieve regression over the dosing interval. Finally, DS-6157a did show tumor regression, but this was in a cell line transfected with additional receptor, so it was not included. Even with inclusion of these 4 additional agents, the fraction of ADCs showing tumor regression at or below the clinical MTD is less than the approved agents (30% versus 100%).

ADC	Target	Payload	Notes	Minimum effective dose in mice	MTD in clinic	Preclinical	Clinical trial
DEDN6526A; RG7636	EDNBR (melanoma)	MMAE	11% PR 32% SD	1 mg/kg	2.4 mg/kg Q3W	Link	Link
DLYE5953A	Ly6E	MMAE	12% PR 54% SD	1 mg/kg	2.4 mg/kg Q3W	Link	Link
Enapotamab vedotin	AXL	MMAE	3 patients with partial response, but number dosed at this level unclear	1 mg/kg in CDX model, 2 mg/kg Q7Dx2 in PDX models	2.2 mg/kg Q3W	Link	Link
RN927C	TROP2	Aur0101	Below MTD. Though MTD not fully explored due to toxicity. 0% ORR. 37.9% SD	0.75 mg/kg	2.4 mg/kg was highest tolerated dose. MTD not determined.	Link	Link
SAR566658	MUC1	DM4	13% PR and 39% SD	1.9 mg/kg	2.4 mg/kg D1, D8 Q3W	Link	Link
Sofituzumab vedotin (TDC)	MUC16	MMAE	35% of patients had best responses of PR or CR	1.5 mg/kg	5.2 mg/kg Q3W	Link	Link
SYD985	HER2	Duocarmycin	33% PR	1 mg/kg	1.2 mg/kg Q3W	Link	Link
DS-6157a	GPR20	DXd	3% PR with 20% shrinkage	3 mg/kg but only in transfected cell line	6.4 mg/kg Q3W	Link	Link
MDX-1203	CD70	Duocarmycin	69% disease stabilization, 2 doses of 38 mg/kg (76 mg/kg total) needed for CR in mice	Some activity after single doses >=4mg/kg. Complete tumor regression after 2 doses of 38 mg/kg.	8 mg/kg Q3W		Link
PCA062	P-cadherin	DM1	DCR = 33.3% in HNSCC, 22.2% eosophageal cancer, 22.6% other tumors.	Stasis at 2.5 mg/kg	3.6 mg/kg Q2W	Link	Link
Sofituzumab vedotin (ADC)	MUC16	MMAE	4% CR, 12%PR, 68% SD	2 mg/kg Q1Wx3, 3 mg/kg	2.4 mg/kg Q3W	Link	Link
ABT-414	EGFR	MMAF	23% SD. High ocular AEs at MTD.	Tumor stasis at 1 mg/kg Q4Dx3 (SCC15). Tumor reduction at 2 mg/kg Q4Dx6 (U87MGde2-7)	3 mg/kg Q3W	Link	Link
AGS16F	ENPP3	MMAF	23% PR at 1.8 mg/kg. Protocol-defined (3.6 mg/kg Q3W) dose not tolerated in multiple doses.	0.5 mg/kg Q4Dx4 (UG-K3)	RP2D 1.8 mg/kg Q3W	Link	Link
ASG-5ME	SLC44A4	MMAE	52% PR or SD	1 mg/kg Q4Dx4	2.7 mg/kg Q3W	Link	Link
BAY1187982	FGFR2	Auristatin W derivative	No responses recorded.	1 mg/kg Q7Dx3	0.2 mg/kg Q3W	Link	Link
Cantuzumab mertansine	CanAg	DM1		12 mg/kg Q1Dx5	3.1 mg/kg Q1W	Link	Link
Glembatumumab vedotin	CR011	MMAE	15%PR, 24% SD	2.5 mg/kg Q4Dx4	1.88 mg/kg Q3W	Link	Link
LOP628	c-KIT	DM1		0.625 mg/kg	0.3 mg/kg	Link	Link
Lorvotuzumab mertansine	CD56	DM1	33.3% CBR for ovarian cancer patients. 21.2% CBR for SCLC patients.	8.5 mg/kg	2.03 mg/kg on 3 consecutive days every 21 days	Link	Link
PF-06650808	NOTCH3	Aur0101	9.7% ORR, 16.7% ORR in breast cancer patients, 21.4% in ER+ breast cancer	1 mg/kg Q4Dx4	2.4 mg/kg Q3W	Link	Link
SGN-75	CD70	MMAF	2.1% CR, 4.3% PR, 43% SD	2 mg/kg Q4Dx4	3 mg/kg Q3W	Link	Link
ABBV-085	LRRC15	MMAE	20% ORR	6 mg/kg Q4Dx6	3.6 mg/kg Q2W	Link	Link
Anetumab ravtansine	Mesothelin	DM4	0.7% CR, 7.4% PR, 44.6% SD	2.7 mg/kg Q3Dx3	6.5 mg/kg Q3W or 2.2 mg/kg Q1W	Link	Link
DMOT4039A	Mesothelin	MMAE	Pancreatic cancer (8% PR, 35% SD), Ovarian cancer (30% PR)	5 mg/kg	2.4 mg/kg Q3W	Link	Link
IMMU-130	CEACAM5	SN-38	29% (PR+SD)	25 mg/kg twice weekly for 4 weeks	16 mg/kg Q2W	Link	Link
PF-06263507	5T4	MMAF	7.7% SD	3 mg/kg Q4Dx4	4.34 mg/kg Q3W	Link	Link
RovaT	DLL3	PBD	18% had confirmed objective response	1 mg/kg Q4Dx3	0.4 mg/kg Q3W	Link	Link
TAK-164	GCC	DGN549	4% ORR, 44% SD	0.76 mg/kg	0.064 mg/kg Q3W	Link	Link
TAK-264	GCC	MMAE	2.6% PR, 7.7% SD	7.5 mg/kg Q1Wx3	1.8 mg/kg Q3W	Link	Link
CMB-401	PEM	Calicheamicin	No PR	Treatment started before tumors were palpable (2-3 days after injection of cells)	0.43 mg/kg Q4Wx7	Link	Link
CMD-193	LewisY	Calicheamicin	Rapid clearance and hepatic uptake led to termination.	~2.3 mg/kg Q4Dx3	0.0973 mg/kg	Link	Link
DHES0815A	HER2	PBD-MA	Discontinued due to safety concerns and narrow therapeutic window.	No tumor reduction in xenograft models. Stasis at 10 mg/kg.	Well tolerated up to 2.4 mg/kg Q3W. Discontinued due to safety concerns and narrow therapeutic window.	Link	Link
MEDI4276	HER2	Tubulysin		3 mg/kg Q1Wx4	0.3 mg/kg Q3W	Link	Link
PF-06647263	EFNA4	Calicheamicin	10% PR, 36.7% SD	0.09 mg/kg Q1Wx2	0.015 mg/kg Q1W	Link	Link
AMG 595	EGFRvIII	DM1	BBB issues. 6% PR, 47% SD	5.6 mg/kg	2 mg/kg Q3W	Link	Link
Bivatuzumab mertansine	CD44v6	DM1	10% PR	2.1 mg/kg Q1Dx5	8.1 mg/kg		Link
SGN-15	BR96	Doxorubicin	3% PR, 36% SD, actually 140 mg/kg in Sjorgen and lowest single dose was 540 mg/kg or 3 doses at 50 mg/kg	140 mg/kg Q4Dx3	23.6 mg/kg Q3W	Link	Link
XMT-1522	HER2	AF-HPA	84.6% SD or better at doses >= 0.43 mg/kg, 1 PR at 0.76 mg/kg	0.67 mg/kg	Highest administered dose 0.765 mg/kg	Link	Link
ADCT-401	PSMA	PBD	12.1% composite response rate (PSA response); 1 unconfirmed PR (3%) and 36.4% SD	0.3 mg/kg	Highest administered dose 0.3 mg/kg	Link	Link
ABBV-176	PRLR	PBD	No clinical response	0.1 mg/kg	Highest administered dose 0.10935 mg/kg (1 patient), other 18 < 0.1 mg/kg	Link	Link
AMG 172	CD27L	DM1	5.4% PR, 16.2% SD	Preclinical data not found	1.6 mg/kg Q2W		Link
DSTP3086S	STEAP1	MMAE	6% PR	Preclinical data not found	2.4 mg/kg Q3W		Link
BAT8001	HER2	Batansine	41% PR 83%DCR	Preclinical data not reported	3.6 mg/kg Q3W		Link
SC-002	DLL3	PBD	14% PR, no CR	Preclinical data not found	Dosing regimen considered to exceed MTD.		Link
SC-003	DPEP3	PBD	4% ORR. Responses not durable.	Preclinical data not found	0.3 mg/kg Q3W		Link
SGN-CD70A	CD70	PBD	15% PR, 5% CR, 30% SD, 20% ORR	Preclinical data not found	0.03 mg/kg Q6W	Link	Link
BMS-986148	Mesothelin	Tubulysin	6% PR, 49% SD	Preclinical data not found	1.2 mg/kg Q3W		Link
MEDI0641	5T4	PBD		1 mg/kg	Clinical trial data not found	Link	
NJH395	HER2	TLR7 agonist	Immunomodulatory payload	Immunomodulatory payload	Immunomodulatory payload	Link	
MEDI-547	EphA2	MMAF	5 patients had an overall response of progressive disease and 1 patient had an overall response of stable disease.	1 mg/kg	Highest administered dose 0.08 mg/kg	Link	Link
CDX-014	TIM-1	MMAE	31% CBR	0.3 mg of ADC (~12 mg/kg) 4 doses over 12 days	Highest administered dose 2 mg/kg	Link	Link
HKT288	Cadherin-6	DM4	Preclinical dosing greater than starting clinical dose (0.3 mg/kg Q3W). Unanticipated neurotoxicity.	2.5 mg/kg	Highest administered dose 0.75 mg/kg	Link	Link
LY3076226	FGFR3	DM4	No responses seen. Study stopped due to pipeline prioritization.	5 mg/kg Q1Wx4	Highest administered dose 5 mg/kg	Link	Link
MLN2704	PSMA	DM1	1/10 patients PR, 4/10 patients SD	30 mg/kg Q2Wx5	Highest administered dose 9.27 mg/kg	Link	Link

Supplemental Method S1:

In Vitro Cell Viability Assay

The *in vitro* viability assay was performed following a previously published protocol. Briefly, mouse breast cancer cells (4T1 and EO771-hHER2) were seeded at 1500 cells/well (in triplicates for each concentration tested) in 96-well black-walled, clear bottom plates one day before the assay started. The cells were allowed to adhere overnight. Titrations of each free payload were replaced daily for 6 days, and the viability was measured using the PrestoBlue Cell Viability Reagent (ThermoFisher Scientific). Cells were incubated with 1:10 dilution of PrestoBlue reagent in RPMI medium for 45 mins and fluorescence (Ex:560/Em:590) of each well was measured using a Biotek Synergy plate reader. The signal was normalized to untreated cells.

Supplemental Method S2:

In Vivo Tumor Xenograft Pharmacodynamic Immunofluorescence Staining

All animal studies were approved and conducted in compliance with the Institutional Animal Care and Use Committee of the University of Michigan (Ann Arbor, MI) and Association for Assessment and Accreditation of Laboratory Animal Care International.

To study intratumoral distribution of ADCs, 6–8-week-old homozygous female nude mice (RRID: 2175030, Foxn1nu/nu, Jackson Laboratories) were injected subcutaneously in the hind limb with ~5e6 NCI-N87 cells (ATCC). When tumors reached 250 mm^3 in size, the mice were injected intravenously via the tail vein with 6.4 mg/kg of Enhertu or 3.6 mg/kg of Kadcyla conjugated to Alexa Fluor 647. The mice were euthanized 72 hrs post administration for tumor resection, and 15 minutes prior to euthanasia, the mice were administered 15 mg/kg Hoechst-33342 intravenously to mark functional blood vessels. Resected tumors were flash frozen in OCT using isopentane chilled on dry ice. Tumor slices were pre-blocked in 0.5% BSA/PBS for 10 minute, and stained *ex vivo* with Phospho-Histone H2A.X (Ser139) primary antibodies (Cell Signaling Technology) or Phospho-Histone H3 ($pSer^{10}$) primary antibodies (Sigma-Aldrich) at 0.15 μg/mL in 0.5% BSA for 30 minutes at room temperature. Slices were washed in PBS for 5 minutes and then incubated with AlexaFluor 555-labeled Goat anti-Rabbit secondary antibody at 2.5 μg/mL in 0.5% BSA/PBS for 30 minutes in the dark at room temperature. Microscopy was performed as described in previous work *(71)*, and image analysis was performed using ImageJ.

Supplemental Method S3:

Calculation of %ID/g from Standard Uptake Value (SUV)

Where literature reported SUV values for tumor uptake of radiolabeled antibodies or small radioactive molecules, the percentage injected dose (%ID/g), assuming 1 g/mL tissue density, was calculated.

$$SUV = \frac{\left(\frac{tumor\ activity}{mL}\right)}{\left(\frac{injected\ activity}{body\ weight}\right)}$$

$$\%ID/g = \frac{\frac{tumor\ activity}{mL} * 100\%}{injected\ activity}$$

%ID/g was therefore calculated from SUV as follows:

$$\%ID/g = \frac{SUV * 100\%}{body\ weight}$$

For an estimated human body weight of 70 kg (70,000 g), $\%ID/g = \frac{SUV}{700}$

Supplemental Method S4:

Calculation of tumor drug concentration from %ID/g

$$\%ID/_g = \frac{Mass\ of\ drug\ in\ the\ tumor * 100\%}{Mass\ of\ drug\ injected * mass\ of\ tumor}$$

$$\%ID/_g = \frac{C_{tumor} * 100\%}{C_{plasma,0} * V_{plasma} * \rho_{tumor}}$$

$$C_{tumor} = \frac{\%ID/_g * C_{plasma,0} * V_{plasma} * \rho_{tumor}}{100\%}$$

$$C_{tumor}(nM) = \frac{\%ID/_g * nmol\ dose * 1000g/L}{100\%}$$

$$C_{tumor}(nM) = \frac{\%ID/_g * \frac{nmol\ dose}{kg} * BW(kg) * 1000g/L}{100\%}$$

$$\frac{C_{tumor}(nM)}{\frac{nmol\ dose}{kg}} = \frac{\%ID/_g * BW(kg) * 1000g/L}{100\%}$$

<u>Definition of terms:</u>

$$C_{tumor} = Concentration\ of\ drug\ in\ tumor$$

$$C_{plasma,0} = Initial\ concentration\ of\ drug\ in\ plasma$$

$$V_{plasma} = Volume\ of\ plasma$$

$$\rho_{tumor} = Density\ of\ tumor$$

$$BW = Body\ Weight$$

<u>Assumptions:</u>

a) $\rho_{tumor} = \rho_{water} = 1\ g/mL$
b) $V_{human\ plasma}$ = 3500 mL
c) $V_{mouse\ plasma}$ = 1.4 mL

Supplemental Figure S1:

DAR 1.6 Sofituzumab vedotin shows greater efficacy than DAR 3.1 Sofituzumab vedotin *in-vivo* at the same payload dose.

With the same antibody and same payload, the lower DAR thiomab agent enabled twice the antibody dose with a similar payload dose, which resulted in twice the clinical response rate. A higher antibody dose is predicted to increase tumor penetration and delivery of payload to a larger fraction of cells per tumor volume, enabling improved efficacy *(30, 61)*.

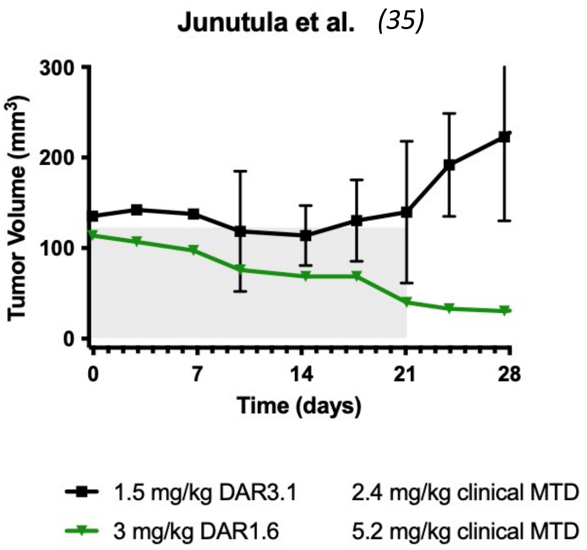

Supplement: Supplementary file 1 — Table S1 Methods S1 to S4 Fig. S1 [file sciadv.adk1894_sm.pdf]
